# Supplementary material for: Human adipose glycerol flux is regulated by a pH gate in AQP10
Source: Nat Commun. 2018 Nov 12;9:4749. doi: 10.1038/s41467-018-07176-z (PMC6232157; doi:10.1038/s41467-018-07176-z)
Supplement: Supplementary file 1 — Supplementary Information [file 41467_2018_7176_MOESM1_ESM.docx]

**Human adipose glycerol flux is regulated by a pH gate in AQP10**

Kamil Gotfryd et al., 2018

Supplementary Information, including

Supplementary Figures 1-10

Supplementary Tables 1-4

Supplementary Fig. 1.


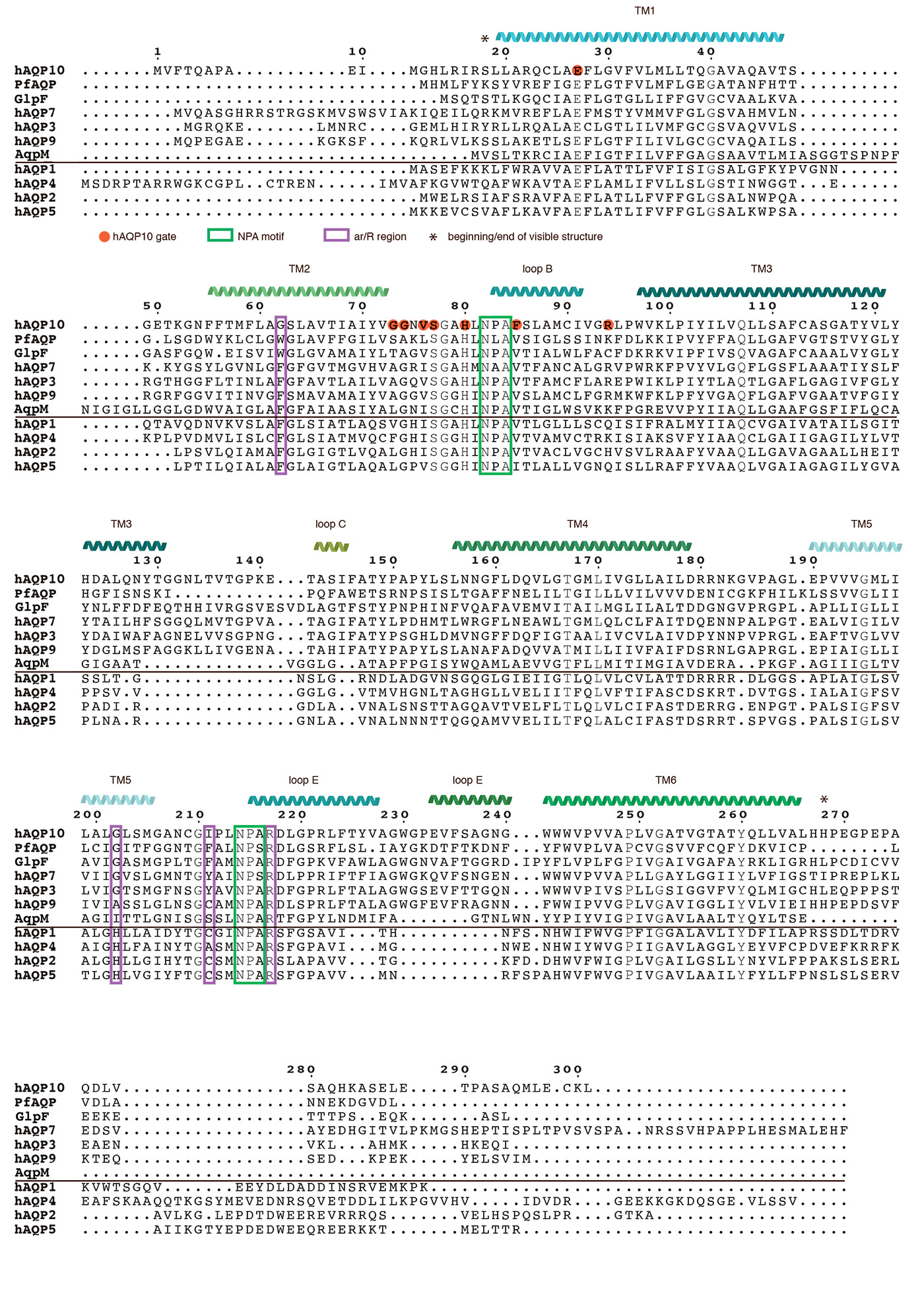


Supplementary Fig. 1. Sequence alignment of human AQP10 and other selected aquaporins. Sequences are numbered starting with the first amino acid in the mature protein. UniProt accession numbers are provided for full-length proteins: hAQP1 (P29972), hAQP2 (P41181), hAQP3 (Q92482), hAQP4 (P55087), hAQP5 (P55064), hAQP7 (O14520), hAQP9 (O43315), hAQP10 (Q96PS8), PfAQP (Q8WPZ6), AqpM (Q9C4Z5), GlpF (P0AER0). NPA and ar/R regions are framed (green and purple boxes, respectively). Residues highlighted in orange circles are involved in the hAQP10 gate.

Supplementary Fig. 2.

**
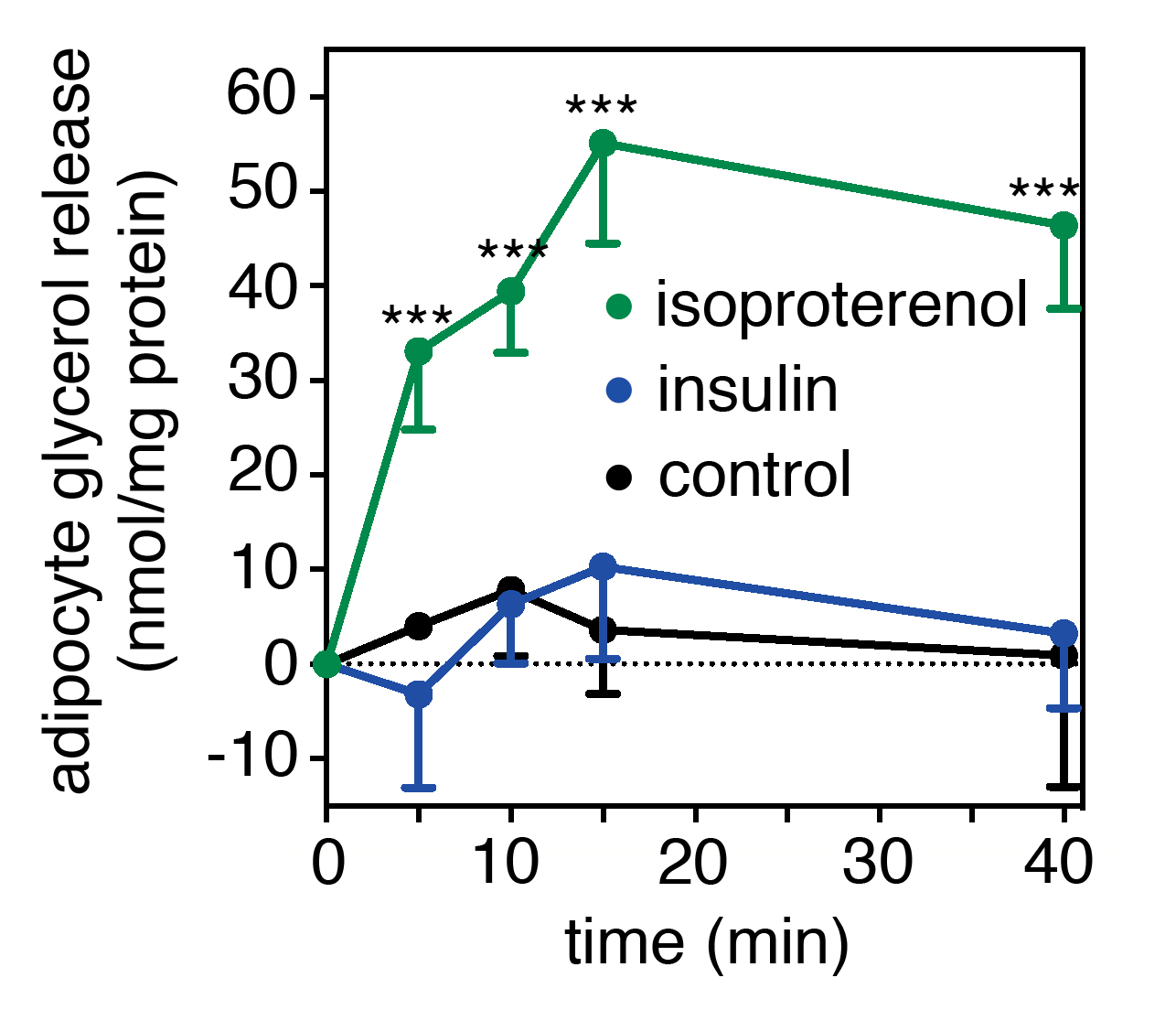
**

Supplementary Fig. 2. Effect of lipolysis on glycerol export from human adipocytes. Glycerol release into the media from human adipocytes under basal (control, black), lipogenic (insulin, blue) and lipolytic (isoproterenol, green) conditions. Results are given as mean ± SEM. ***, P<0.001 *vs.* control and insulin (ANOVA followed by Newman-Keuls’s Q test; *N=*4).


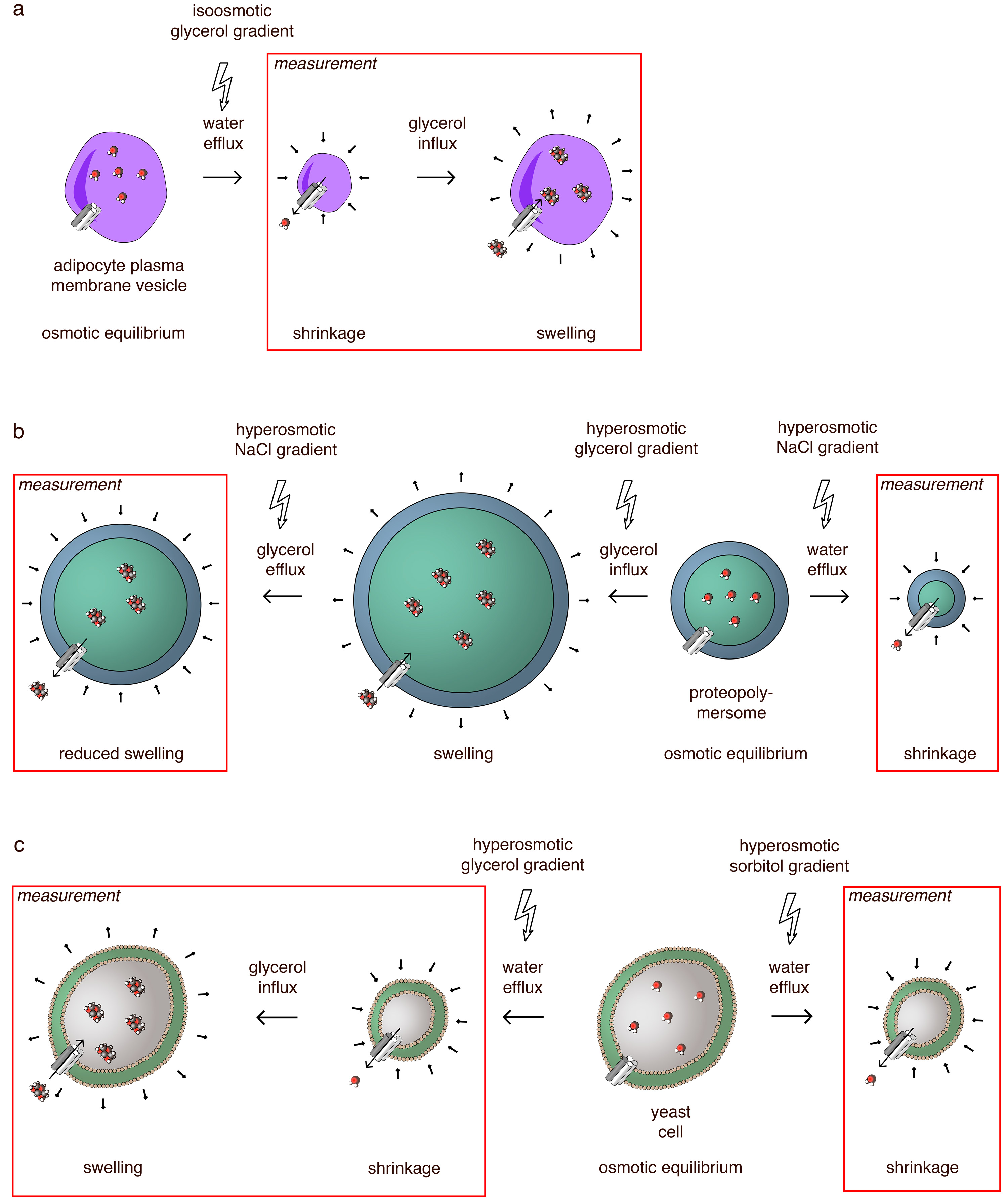
Supplementary Fig. 3.

Supplementary Fig. 3. Overview of the three activity assays used for functional characterization of AQP-dependent fluxes. (a) Human adipocyte plasma membrane vesicle-based assay. Water and glycerol permeabilities of vesicles were induced by isosmotic glycerol gradient and measured exploiting stopped-flow light scattering. Initially there is an increase in light scattering resulting from vesicle shrinkage caused by osmotic water efflux followed by a slower decrease resulting from vesicle swelling due to glycerol entry triggering water influx. (b) Proteopolymersome-bassed assay. Human AQPs were reconstituted into polymersomes and formed proteopolymersomes were exposed to either hyperosmotic NaCl gradient (to allow water efflux) or hyperosmotic glycerol gradient followed by NaCl osmotic stress to induce glycerol efflux. Permeation was measured using stopped-flow light scattering. (c) Intact yeast cell-based assay. Cells overexpressing respective human AQPs were preloaded with nonfluorescent dye that is intracellularly converted into impermeable fluorescent form. Water and glycerol permeabilities of cells were induced by hyperosmotic sorbitol or glycerol gradients, respectively and measured employing stopped-flow fluorescence.

Supplementary Fig. 4.

**
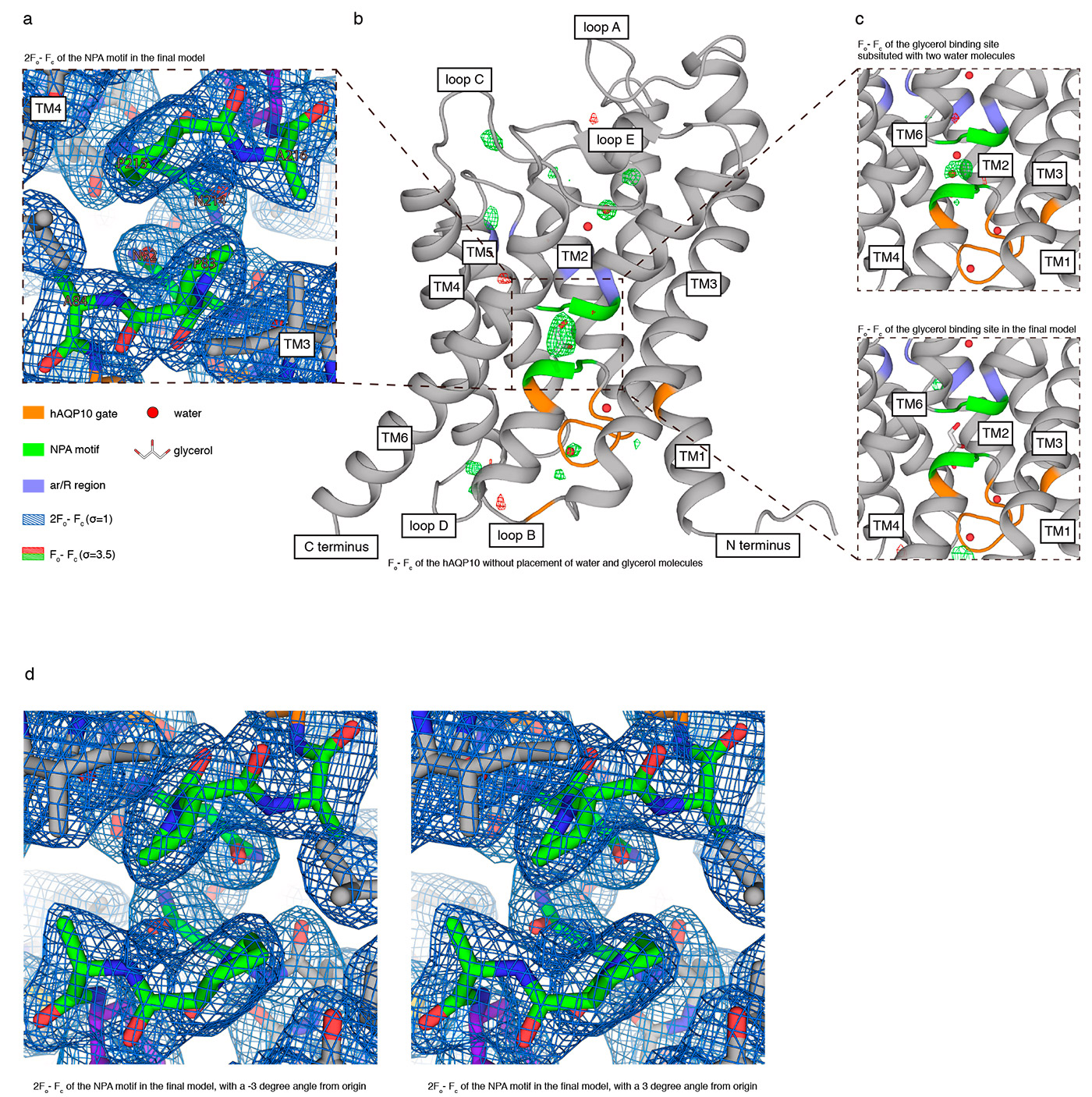
**

Supplementary Fig. 4. Electron density. (a) Close view of the NPA region of chain D with the final 2F_O_-F_C_ electron density shown as blue mesh (σ=1.0). The protein is colored as in Fig. 2c. (b) Side view of chain D with a F_O_-F_C_ difference map (green and red mesh for positive and negative density, σ=3.5) calculated using the final model without water and glycerol molecules. The protein is colored as in Fig. 2f. For clarity the electron density is shown around the water and glycerol-conducting pore only. The data suggest that a single glycerol molecule is located in the pore. (c) Top panel: side view of chain D with a F_O_-F_C_ difference map (σ=3.5) calculated using the final model placing two ordered water molecules in the larger green density shown in Supplementary Fig. 4b. Bottom panel: side view of chain D with a F_O_-F_C_ difference map (σ=3.5) calculated using the final model placing a single glycerol molecule in the larger green density shown in Supplementary Fig. 4b. (d) Stereo view of NPA motif visualized in (a) +/- 3 degrees from origin.

Supplementary Fig. 5.


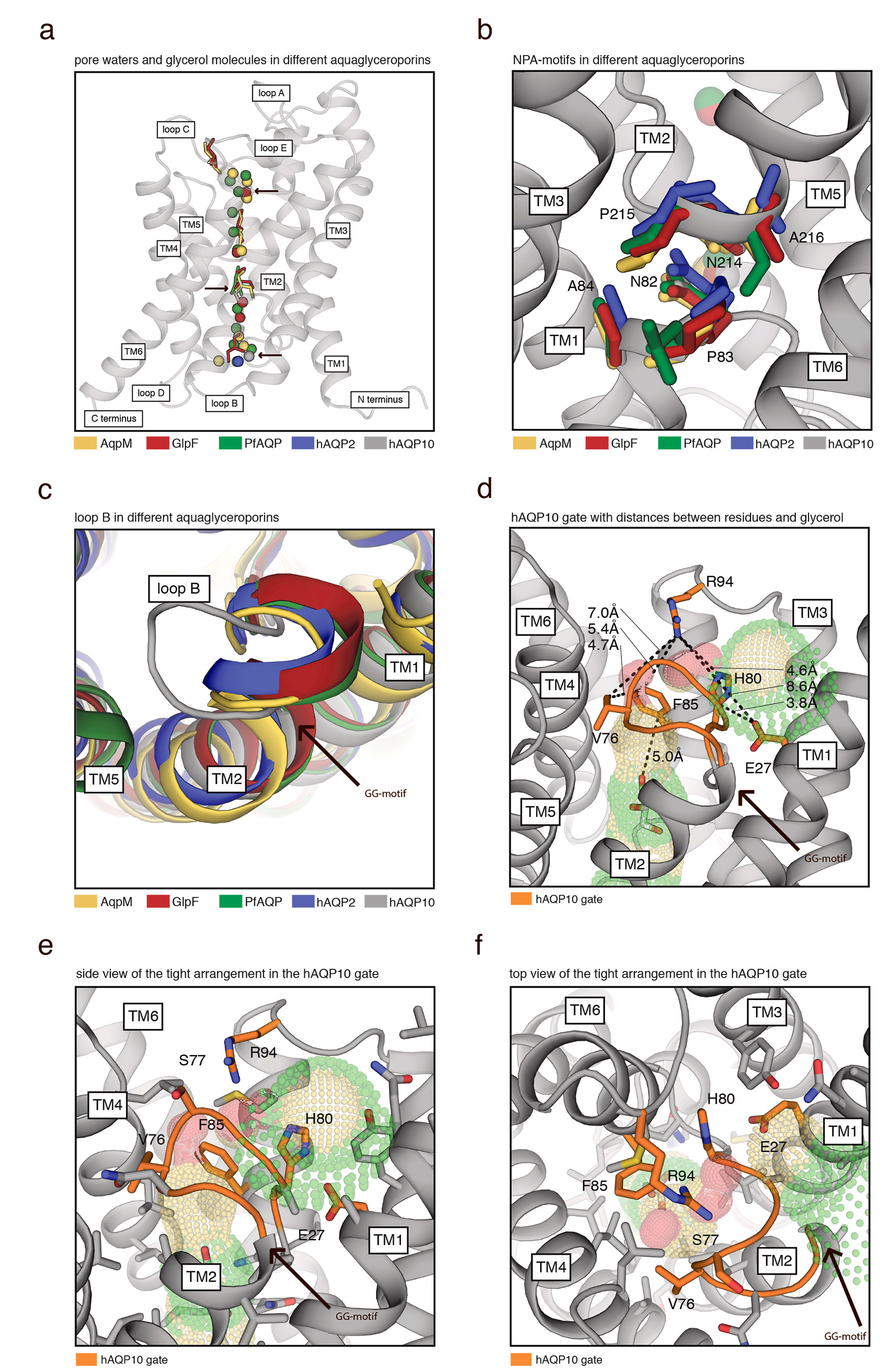


# Supplementary Fig. 5. Unique structural features of hAQP10. (a) hAQP10 (chain A, gray) with pore waters and glycerol molecules from different structurally determined aquaglyceroporins (colors identical as in Fig. 2e). Arrows indicate pore waters and glycerol in hAQP10. (b) Close-view of the NPA-motifs of hAQP10 (chain A, gray) compared to different structurally determined aquaglyceroporins (colors identical as in Fig. 2e). Glycerol molecules in the obtained structures are shown as spheres in equivalent colors. (c) Overlay of loop B from different structurally determined aquaglyceroporins (colors identical as in Fig. 2e). (d) Distances between residues and glycerol molecule in the hAQP10 gate region. Distances were estimated between atoms within the shortest reach between residues. (e) Close-view of the hAQP10 gate with residues shown within 3.5 Å of the HOLE pore. (f) Alternative close-view of the hAQP10 gate with residues shown within 3.5 Å of the HOLE pore.

# Supplementary Fig. 6.


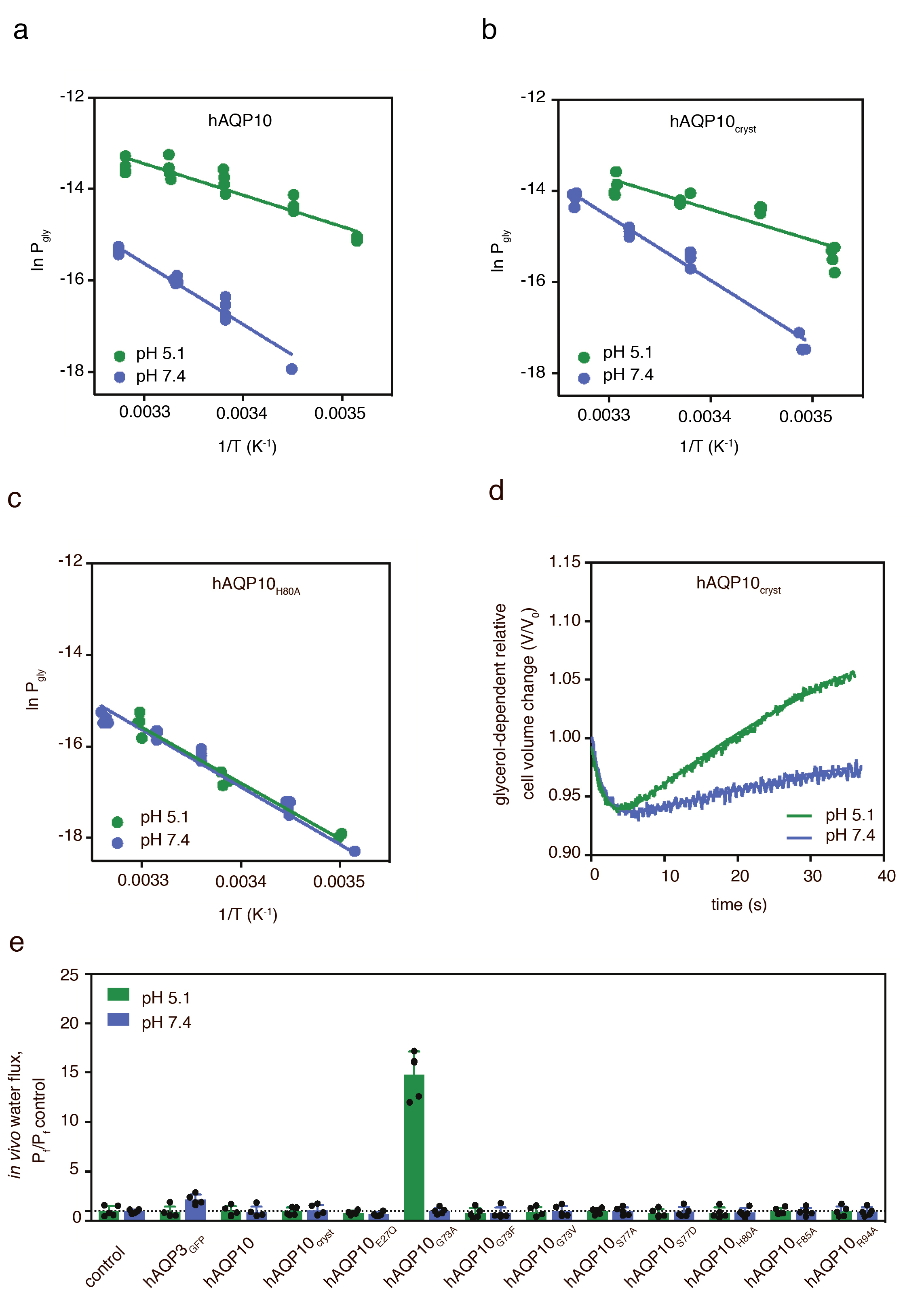


Supplementary Fig. 6. Functional characterization of human AQP10 in vivo. (a-c) Arrhenius plots for estimation of the activation energy (E_a_) for glycerol permeability in cells expressing hAQP10 forms measured at pH 5.1 (green) and pH 7.4 (blue). Results from *N*=3 independent experiments are shown. (d) Representative time course of the relative cell volume (V/V_0_) changes after glycerol hyperosmotic shock at pH 5.1 (green) and 7.4 (blue) in hAQP10_cryst_ expressing intact yeast cells. After the fast water efflux, glycerol influx *via* hAQP10_cryst_ induces cell reswelling. (e) Water permeability (P_f_) ratio of yeast cells expressing hAQP3_GFP_ or hAQP10 forms measured at pH 5.1 (green) and pH 7.4 (blue). Results are normalized to P_f_ of the control strain (without plasmid) at the respective pH. Data for hAQP10_F85V_ are not shown as P_f_ was unmeasurable (rapid glycerol entry masking the water outflow signal). Results are given as mean ± SD of *N*=5 independent experiments.

Supplementary Fig. 7.


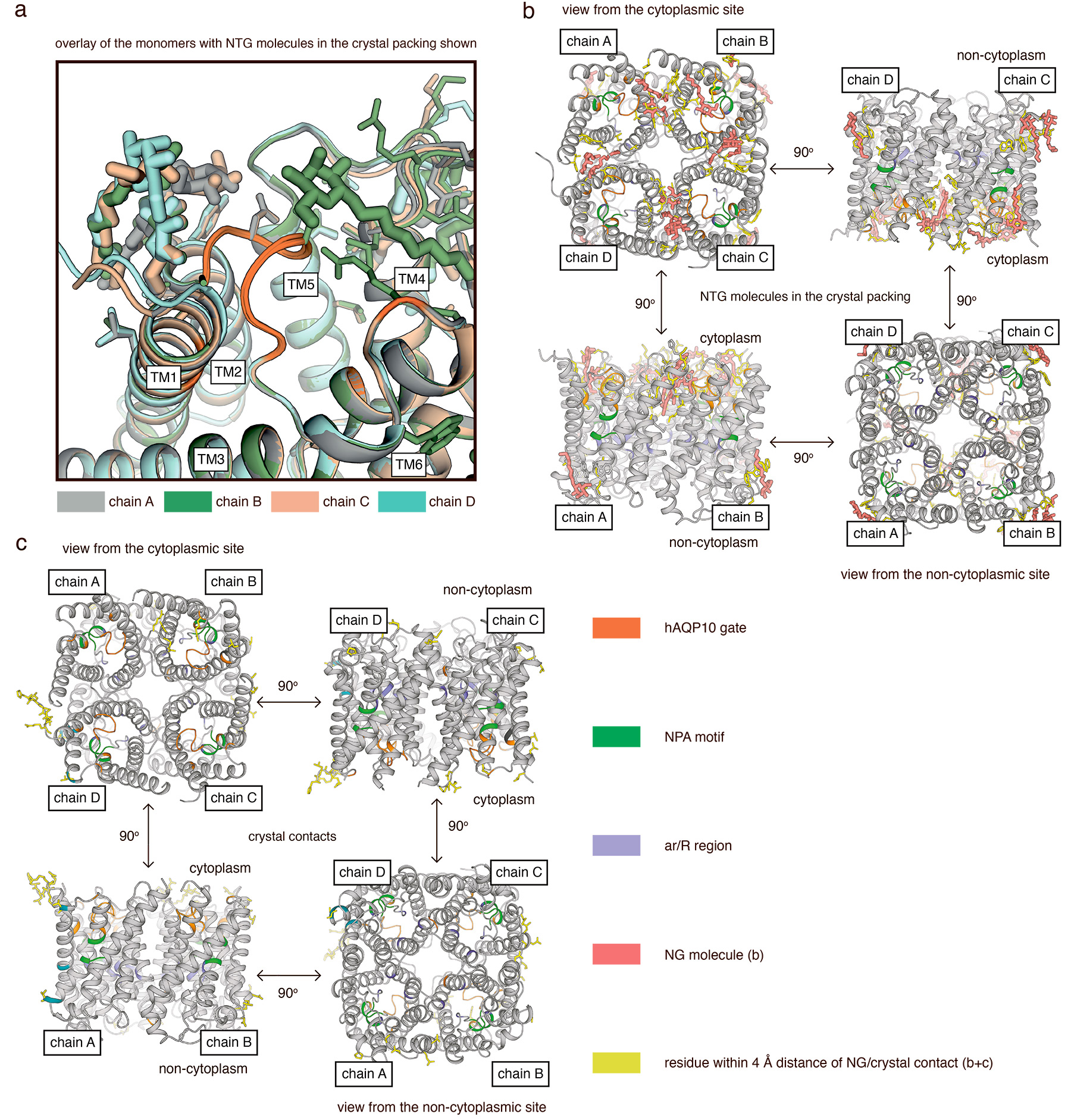


Supplementary Fig. 7. In crystallo interactions of human AQP10. (a) Close view of the hAQP10 gate region with all four monomers (and associated n-nonyl-β-D-glucopyranoside, NG, detergent molecules in equivalent colors as the monomers) superimposed. The NG molecules and residues within 4 Å from the detergent molecules are shown as sticks. Two NG molecules are observed between two sets of monomers and three NG molecules between the remaining two sets. An additional NG molecule is observed in the membrane interface. (b) Overall views with associated NG molecules are shown as pink thick sticks, and with interacting residues within 4 Å in yellow. The ar/R, NPA and hAQP10 gate filters are shown in purple, green and orange, respectively. (c) Residues involved in crystal contacts (within 4 Å of an adjacent tetramers) are shown in yellow. The ar/R, NPA and hAQP10 gate filters are shown in purple, green and orange, respectively. The gate region is in general not involved in crystal packing with adjacent tetramers.

Supplementary Fig. 8.


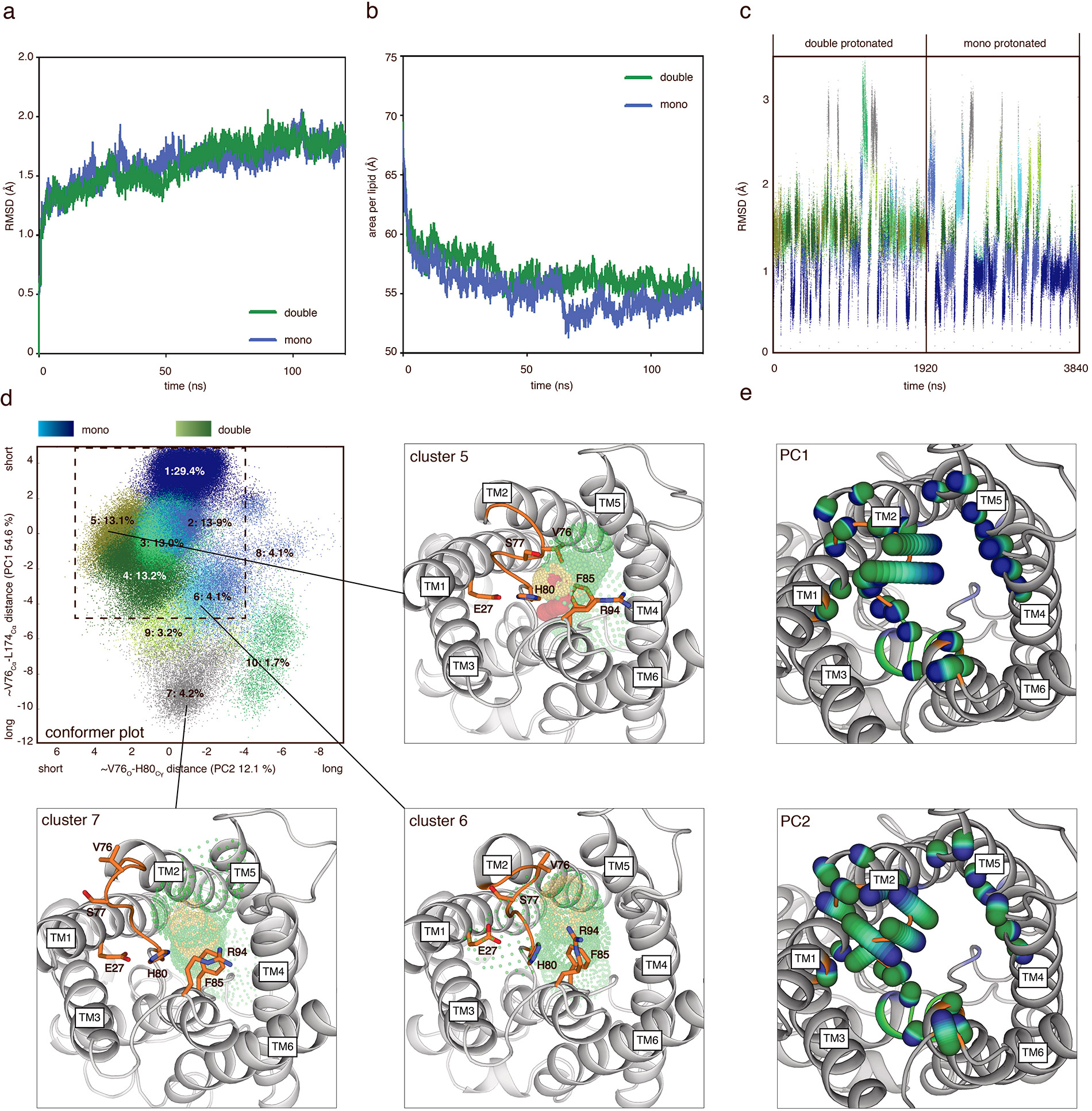


# Supplementary Fig. 8. Overall behavior of the molecular dynamics simulations. (a and b) Root mean square displacement (RMSD, panel a) and area per lipid (panel b) for representative mono (blue) and double (green) protonated simulations. (c) RMSD plot of identified clusters based on 28 selected residues of the cytoplasmic region of the combined mono (blue palette) and double (green palette) protonated simulations. Each monomer is put in sequence (A, B, C, D) for the 4 simulation cases for the respective protonation state (in total 16 monomers for each simulation). The majority of the single protonated frames represent structures similar to the crystal structures, while the double protonated quickly changes conformation to a more open state. (d and e) Principal component (PC) analysis of the total combined trajectory for the mono and double protonated simulations. The first and second principal component accounts for 54.6 % and 12.1 % of the total variance, respectively. (d) The full conformer plot with clusters labeled in blue and green colors for mono and double protonated simulations and with the % of frequency of each cluster indicated. The dotted square illustrates the equivalent plot as shown in Fig. 4a. The equivalent close views as shown in Fig. 4a for the less populated clusters #5-7 surround the conformer plot. (e) The extrapolated Cartesian coordinates of the PCs showing the identified motions. Simplified, PC1 and PC2 represent the pore width distances between V76 and L174 and between V76 and H80 (see also Supplementary Fig. 9).

Supplementary Fig. 9.

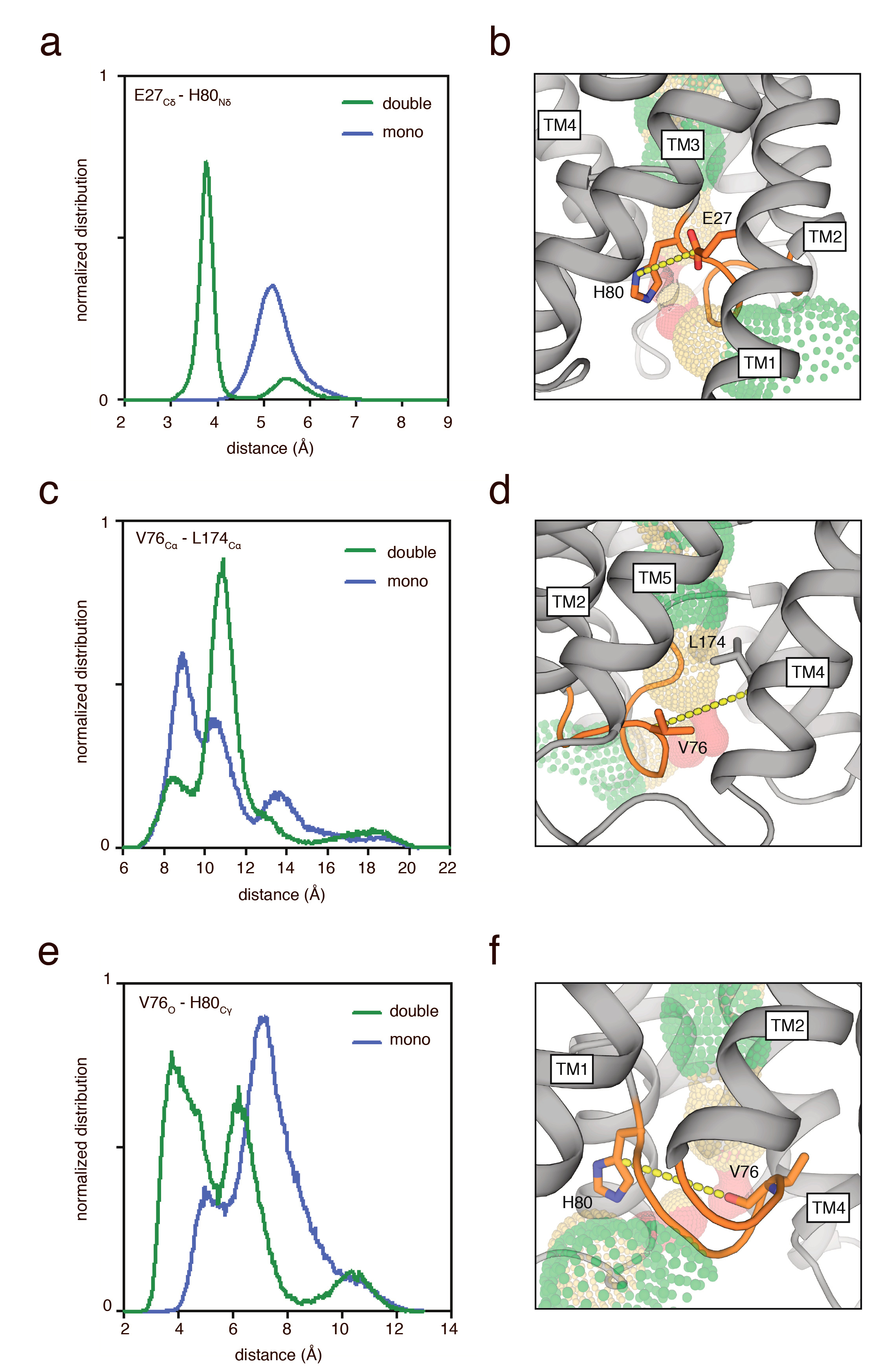


Supplementary Fig. 9. Difference between mono and double protonated molecular dynamics simulations. (a, c and e) Histograms for the distribution of distances observed between E27_Cδ_-H80_Nδ_, V76_Cα_-L174_Cα_ and V76_O_-H80_Cγ_, respectively. Double protonated in green and mono protonated in blue. (b, d and f) The equivalent distances (shown in yellow) as observed in the crystal structure (chain A), colored as in Fig. 2e.

Supplementary Fig. 10.


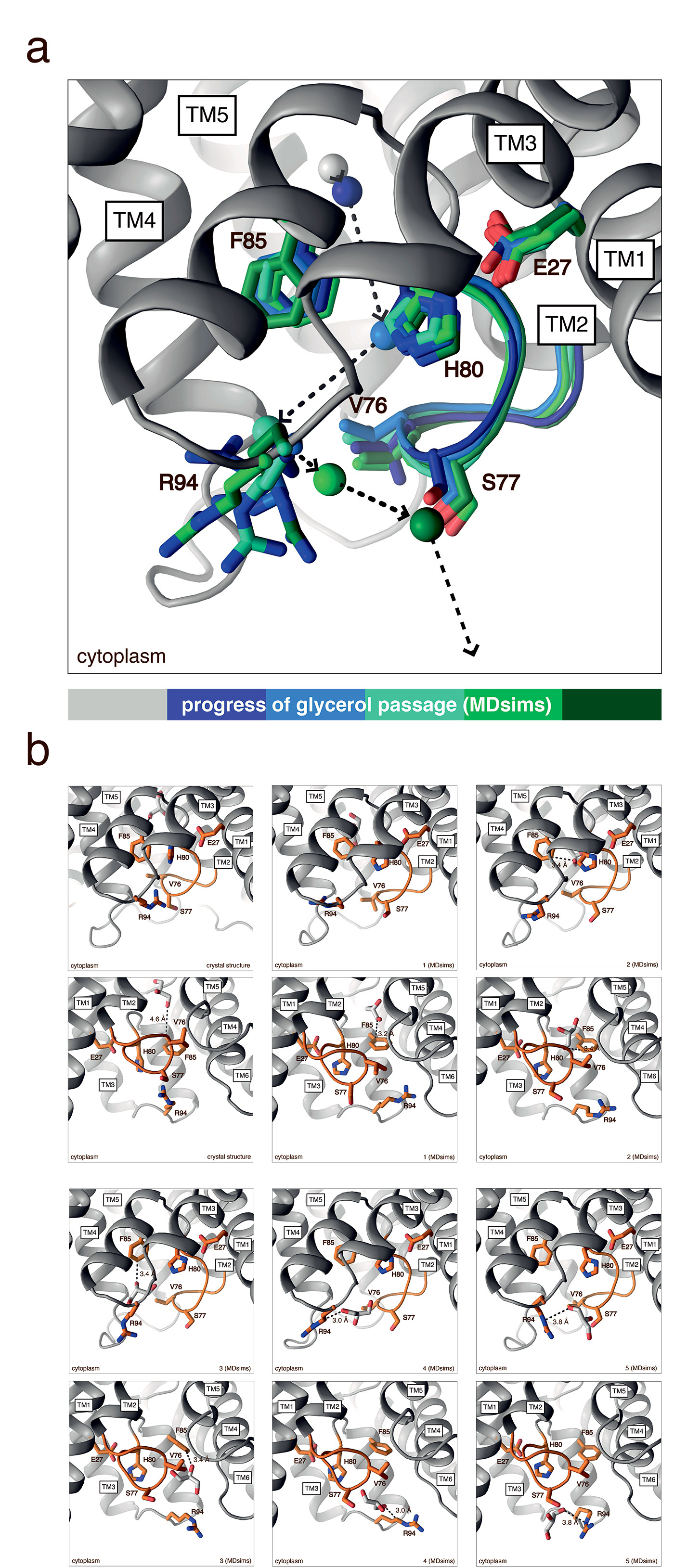


Supplementary Fig. 10. Representative snapshots of a glycerol passage event across the gate region observed in MD simulations with H80 being double protonated. (a) The crystal structure with its glycerol molecule location is shown in gray. The color palette, from blue to green for selected residues and the glycerol molecules, demonstrate the glycerol passage progress observed during the simulation. (b) Separate views of the individual snapshots used in (a) from two different angles (0 degrees and 180 degrees compared to the view in (a)).

# Supplementary Table 1. Summary of recombinant protein constructs used in the study.

| Assay | Construct | Primer names and sequences | C-terminus (features) | Expression plasmid | *S. cerevisiae* expression strain |
| --- | --- | --- | --- | --- | --- |
| creation of C-terminally TEV-GFP-His_10_-fused AQPs | hAQP_GFP_ | hAQP_GFP_ FW: 5’-GAAAATTTGTATTTTCAAAGTCAATTTATGTCTAAAGGTGAAGAATTATTCACT-3’  hAQP_GFP_ RV: 5’-CTTCAATGCTATCATTTCCTTTGATATTGGATCATTCAATGGTGATGGTGATGGTGATGGTGATGGTGTTTGTACAATTCATCCATACCA-3’ | TEV-GFP- His_10_ | pPAP2259 | PAP  1500 |
| functional characterization in proteopolymersomes | hAQP2_GFP_ | hAQP2_GFP_ FW:  5’-ACACAAATACACACACTAAATTACCGGATCAATTCTAAGATAATTATGTGGGAATTGAGATCCATA-3’  hAQP2_GFP_ RV: 5’-AAATTGACTTTGAAAATACAAATTTTCAGCCTTGGTACCTCTAGGTA-3’ | GFP-TEV-His_10_ | pPAP2259 | PAP  1500 |
|  | hAQP3_GFP_ | hAQP3_GFP_ FW:  5’-ACACAAATACACACACTAAATTACCGGATCAATTCTAAGATAATTATGGGTAGACAAAAAGAATTAGT-3’  hAQP3_GFP_ RV:  5’-AAATTGACTTTGAAAATACAAATTTTCAATTTGTTCCTTGTGTTTTACAT-3’ |  |  |  |
|  | hAQP7_GFP_ | hAQP7_GFP_ FW:  5’-ACACAAATACACACACTAAATTACCGGATCAATTCTAAGATAATTATGGTCCAAGCCTCCGGTC-3’  hAQP7_GFP_ RV: 5’-AAATTGACTTTGAAAATACAAATTTTCAAAATGTTCCAAGGCCATAGATT-3’ |  |  |  |
|  | hAQP9_GFP_ | hAQP9_GFP_ FW: 5’-ACACAAATACACACACTAAATTACCGGATCAATTCTAAGATAATTATGCAACCAGAAGGTGCCG-3’  hAQP9_GFP_ RV: 5’-AAATTGACTTTGAAAATACAAATTTTCCATAATTACTGACAATTCGTACTTT-3’ |  |  |  |
|  | hAQP10_GFP_ | hAQP10_GFP_ FW: 5’-ACACAAATACACACACTAAATTACCGGATCAATTCTAAGATAATTATGGTTTTTACACAAGCACCTG-3’  hAQP10_GFP_ RV: 5’-AAATTGACTTTGAAAATACAAATTTTCTAACTTGCATTCCAACATTTGT-3’ |  |  |  |
|  | hAQP10 | hAQP10 FW: 5’-TGCTATCATTTCCTTTGATATTGGATCATTCAATGGTGATGGTGATGGTGATGGTGTAACTTGCATTCCAACATTTGTGC-3’  hAQP10 RV: 5’-ACACAAATACACACACTAAATTACCGGATCAATTCTAAGATAATTATGGGTCACTTGAGAATCAGA-3’ | His_8_ |  |  |
|  | hAQP10_cryst_ | hAQP10_cryst_ FW: 5’-ACACAAATACACACACTAAATTACCGGATCAATTCTAAGATAATTATGGGTCACTTGAGAATCAGA-3’  hAQP10_cryst_ RV: 5’-TCAATGCTATCATTTCCTTTGATATTGGATCATTCAATGGTGATGGTGATGGTGATGGTGGTCTTGGGCAGGTTCTGGA-3’ |  |  |  |
|  | hAQP10_S77A_ | hAQP10_S77A_ FW: 5’-GTTGGTGGTAATGTCGCTGGTGCACATTTAAAC-3’  hAQP10_S77A_ RV: 5’-GTTTAAATGTGCACCAGCGACATTACCACCAAC-3’ |  |  |  |
|  | hAQP10_H80A_ | hAQP10_H80A_ FW: 5’-GTAATGTCTCTGGTGCAGCTTTAAACCCAGCCTTC-3’  hAQP10_H80A_ RV: 5’-GAAGGCTGGGTTTAAAGCTGCACCAGAGACATTAC-3’ |  |  |  |
|  | hAQP10_F85A_ | hAQP10_F85A_ FW: 5’-CATTTAAACCCAGCCGCCTCATTGGCTATGTGTATTG-3’  hAQP10_F85A_ RV: 5’-CAATACACATAGCCAATGAGGCGGCTGGGTTTAAATG-3’ |  |  |  |
|  | hAQP10_R94A_ | hAQP10_R94A_ FW: 5’-CTATGTGTATTGTCGGTGCATTGCCATGGGTAAAG-3’  hAQP10_R94A_ RV: 5’-CTTTACCCATGGCAATGCACCGACAATACACATAG-3’ |  |  |  |
| crystallization | hAQP10_cryst_ | hAQP10_cryst_ FW: 5’-ACACAAATACACACACTAAATTACCGGATCAATTCTAAGATAATTATGGGTCACTTGAGAATCAGA-3’  hAQP10_cryst_ RV: 5’-TCAATGCTATCATTTCCTTTGATATTGGATCATTCAATGGTGATGGTGATGGTGATGGTGGTCTTGGGCAGGTTCTGGA-3’ | His_8_ | pPAP2259 | PAP  1500 |
| functional characterization in intact yeast cells | hAQP3 | hAQP3 FW: 5’- GGACTAGTCCTATGGGTCGACAGAAGGAGCTGGTGTCC-3’  hAQP3 RV: 5’- CCATCGATGGCGATCTGCTCCTTGTGCTTCACATGGGC-3’ | GFP | pUG  35 | YSH  1770 |
|  | hAQP10 | hAQP10 FW:  5’-ATACATAGATACAATTCTATTACCCCCATCCATACTAAGATAATT ATGGTTTTTACACAAGCACCTG-3’  hAQP10 RV: 5’-ACAACACCAGTGAATAATTCTTCACCTTTAGACATTCATAACTTGCATTCCAACATTTG-3’ | − |  |  |
|  | hAQP10_cryst_ | hAQP10_cryst_ FW:  5’-ATACATAGATACAATTCTATTACCCCCATCCATACTAAGATAATTATGGGTCACTTGAGAATCAGA-3’  hAQP10_cryst_ RV: 5’-ACAACACCAGTGAATAATTCTTCACCTTTAGACATTCAGTCTTGGGCAGGTTCTGGA-3’ |  |  |  |
|  | hAQP10_E27Q_ | hAQP10_E27Q_ FW: 5’-TGTTTAGCCCAATTTTTGGGTGT-3’  hAQP10_E27Q_ RV: 5’-ACACCCAAAAATTGGGCTAAACA-3’ |  |  |  |
|  | hAQP10_G73A_ | hAQP10_G73A_ FW: 5’-CTATAGCAATCTACGTTGCTGGTAATGTCTCTGGTGCAC-3’  hAQP10_G73A_ RV: 5’-GTGCACCAGAGACATTACCAGCAACGTAGATTGCTATAG-3’ |  |  |  |
|  | hAQP10_G73F_ | hAQP10_G73F_ FW: 5’-CTATAGCAATCTACGTTTTTGGTAATGTCTCTGGTGCAC-3’  hAQP10_G73F_ RV: 5’-GTGCACCAGAGACATTACCAAAAACGTAGATTGCTATAG-3’ |  |  |  |
|  | hAQP10_G73V_ | hAQP10_G73V_ FW: 5’-CTATAGCAATCTACGTTGTTGGTAATGTCTCTGGTGCAC-3’  hAQP10_G73V_ RV: 5’-GTGCACCAGAGACATTACCAACAACGTAGATTGCTATAG-3’ |  |  |  |
|  | hAQP10_S77A_ | hAQP10_S77A_ FW: 5’-GTTGGTGGTAATGTCGCTGGTGCACATTTAAAC-3’  hAQP10_S77A_ RV: 5’-GTTTAAATGTGCACCAGCGACATTACCACCAAC-3’ |  |  |  |
|  | hAQP10_S77D_ | hAQP10_S77D_ FW: 5’-GTTGGTGGTAATGTCGATGGTGCACATTTAAAC-3’  hAQP10_S77D_ RV: 5’-GTTTAAATGTGCACCATCGACATTACCACCAAC-3’ |  |  |  |
|  | hAQP10_H80A_ | hAQP10_H80A_ FW: 5’-GTAATGTCTCTGGTGCAGCTTTAAACCCAGCCTTC-3’  hAQP10_H80A_ RV: 5’-GAAGGCTGGGTTTAAAGCTGCACCAGAGACATTAC-3’ |  |  |  |
|  | hAQP10_F85A_ | hAQP10_F85A_ FW: 5’-CATTTAAACCCAGCCGCCTCATTGGCTATGTGTATTG-3’  hAQP10_F85A_ RV: 5’-CAATACACATAGCCAATGAGGCGGCTGGGTTTAAATG-3’ |  |  |  |
|  | hAQP10_F85V_ | hAQP10_F85V_ FW: 5’-AACCCAGCCGTCTCATTGGCTA-3’  hAQP10_F85V_ RV: 5’- TAGCCAATGAGACGGCTGGGTT-3’ |  |  |  |
|  | hAQP10_R94A_ | hAQP10_R94A_ FW: 5’-CTATGTGTATTGTCGGTGCATTGCCATGGGTAAAG-3’  hAQP10_R94A_ RV: 5’-CTTTACCCATGGCAATGCACCGACAATACACATAG-3’ |  |  |  |

h: Homo sapiens sequences (codon optimized or native sequences, see Methods)

GFP: green fluorescent protein

cryst: crystallization construct (N- (Δ1-10) and C-termini (Δ277-301) truncated human wild-type AQP10)

TEV: Tobacco Etch Virus protease cleavage site

His_10_: decahistidine tag

His_8_: octahistidine tag

turquoise: homologous recombination sequence

red: *S. cerevisiae PMR1* Kozak sequence

green: Tobacco Etch Virus protease cleavage site-encoding sequence

orange: STOP codon

blue: sequence encoding respective polyhistidine tag

underlined: template-specific part

orange: *SpeI*/*ClaI* restriction enzyme sites

# Supplementary Table 2. Summary of activities of human GFP-fused aquaporins and human AQP10 forms reconstituted into polymersomes (see Figs. 1c and 3a for the equivalent fluxes at the respective pH). Hydrodynamic diameters of the polymeric vesicles were determined by dynamic light scattering (DLS). k_i_ values (s^−1^) were determined by fitting the light scattering signal to a first order exponential function. P_f_ values (m s^−1^) represent calculated water permeabilities. Δdia (nm) displays change in heterodynamic diameter after incubation with glycerol as measured by DLS. Δk_i_ values (s^−1^) show the change in k_i_ values after pre-exposure to glycerol.

| Construct | pH | Hydrodynamic  diameter (nm) | k_i_  (s^−1^) | P_f_  (× 10^−6^ m s^−1^) | Δdia  (nm) | Δk_i_  (s^−1^) |
| --- | --- | --- | --- | --- | --- | --- |
| empty  polymersomes | 7.4 | 140 ± 20 | 190 | 685 ± 98 | − | − |
| hAQP2_GFP_ | 5.5 | 134 ± 9 | 1651 | 5698 ± 909 | -31 | -508 |
|  | 7.4 | 138 ± 11 | 1757 | 6247 ± 1086 | -17 | -330 |
| hAQP3_GFP_ | 5.5 | 158 ± 9 | 1915 | 7793 ± 831 | -63 | 124 |
|  | 7.4 | 133 ± 10 | 1791 | 6134 ± 839 | 119 | 651 |
| hAQP7_GFP_ | 5.5 | 130 ± 10 | 2273 | 7610 ± 809 | -16 | -108 |
|  | 7.4 | 122 ± 8 | 2157 | 6676± 669 | 15 | 1020 |
| hAQP9_GFP_ | 5.5 | 124 ± 12 | 2232 | 7127± 1475 | -28 | 30 |
|  | 7.4 | 124 ± 9 | 2266 | 7236 ± 1182 | 14 | 969 |
| hAQP10_GFP_ | 5.5 | 90 ± 10 | 2386 | 5530 ± 1329 | 86 | 1115 |
|  | 7.4 | 115 ± 11 | 2266 | 6711 ± 1264 | -1 | 109 |
| hAQP10 | 5.5 | 170 ± 28 | 2005 | 8778 ± 2444 | 16 | 819 |
|  | 7.4 | 197 ± 20 | 2267 | 11500 ± 2090 | 31 | -119 |
| hAQP10_cryst_ | 5.5 | 182 ± 24 | 2157 | 10112 ± 2274 | 0 | 870 |
|  | 7.4 | 173 ± 15 | 1914 | 8530 ± 1303 | 24 | -315 |
| hAQP10_S77A_ | 5.5 | 137 ± 15 | 1906 | 6725 ± 1434 | 22 | 530 |
|  | 7.4 | 143 ± 16 | 1755 | 6462 ± 1741 | -6 | -250 |
| hAQP10_H80A_ | 5.5 | 152 ± 18 | 1561 | 6112 ± 1392 | -19 | -186 |
|  | 7.4 | 183 ± 46 | 1610 | 7588 ± 2864 | -31 | 67 |
| hAQP10_F85A_ | 5.5 | 136 ± 18 | 1561 | 5592 ± 1228 | -6 | -186 |
|  | 7.4 | 181 ± 46 | 1610 | 7563 ± 2806 | -9 | 67 |
| hAQP10_R94A_ | 5.5 | 142 ± 11 | 1557 | 5693 ± 1125 | 15 | -127 |
|  | 7.4 | 143 ± 33 | 1682 | 6196 ± 2126 | 7 | 399 |

# Supplementary Table 3. pKa values and Hill coefficients obtained for glycerol permeability pH-dependence measurements performed using *S. cerevisiae*-based in vivo transport assay. Results are given as mean ± SD of at least *N*=3 independent experiments.

| Construct | pKa | Hill coefficient |
| --- | --- | --- |
| hAQP3_GFP_ | 5.97 ± 0.18 | 1.75 ± 0.12 |
| hAQP10 | 5.92 ± 0.20 | 1.7 ± 0.22 |
| hAQP10_cryst_ | 6.00 ± 0.13 | 2.0 ± 0.15 |
| hAQP10_H80A_ | − | − |

# Supplementary Table 4. Glycerol permeability (P_gly_; cm s^−1^) and activation energy (E_a_; kcal mol^−1^) values obtained for pH-dependence transport measurements performed using *S. cerevisiae*-based in vivo assay. Results are given as mean ± SD of *N*=3-6 independent experiments.

| Construct | pH 5.1 | | pH 7.4 | |
| --- | --- | --- | --- | --- |
|  | P_gly_ (23 ºC)  cm s^−1^ | E_a_  kcal mol^−1^ | P_gly_ (23 ºC)  cm s^−1^ | E_a_  kcal mol^−1^ |
| control  (empty pUG35) | 6.69 × 10^−9^ ± 0.34 | 26.0 ± 2.1 | 11.9 × 10^−8^ ± 2.20 | 25.2 ± 2.0 |
| hAQP3_GFP_ | 6.13 × 10^−8^ ± 0.60 | 20.3 ± 1.8 | 1.87 × 10^−6^ ± 0.91 | 10.1 ± 1.8 |
| hAQP10 | 1.40 × 10^−6^ ± 0.19 | 13.0 ± 1.2 | 6.03 × 10^−8^ ± 1.20 | 26.6 ± 1.9 |
| hAQP10_cryst_ | 1.40 × 10^−6^ ± 0.07 | 13.6 ± 1.7 | 9.19 × 10^−8^ ± 2.60 | 28.0 ± 1.2 |
| hAQP10_H80A_ | 8.27 × 10^−8^ ± 0.69 | 24.2 ± 1.6 | 9.90 × 10^−8^ ± 0.88 | 25.0 ± 1.6 |
